# Association of Metabolic Dysfunction-Associated Steatotic Liver Disease (MASLD) With an Increased Risk of Congestive Heart Failure in Hospitalized Patients With Cirrhosis: A Propensity Score-Matched Analysis

**DOI:** 10.7759/cureus.62441

**Published:** 2024-06-15

**Authors:** Derek Ugwendum, Mouhanad Mohamed, Yazan A Al-Ajlouni, Nso Nso, Basile Njei

**Affiliations:** 1 Department of Internal Medicine, Richmond University Medical Center (Affiliated with Mount Sinai Health System and Icahn School of Medicine at Mount Sinai), New York, USA; 2 Department of Medicine, Brown University, Providence, USA; 3 Department of Physical Medicine and Rehabilitation, Montefiore Medical Center, Wakefield Campus, New York, USA; 4 Department of Cardiovascular Disease, University of Chicago, Chicago, USA; 5 Department of Medicine, Yale School of Medicine, New Haven, USA

**Keywords:** metabolic dysfunction-associated steatotic liver disease (masld), metabolic dysfunction-associated steatotic liver disease, in-hospital mortality, cirrhosis, hospitalized patients, congestive heart failure, non-alcoholic fatty liver disease

## Abstract

Introduction: Metabolic dysfunction-associated steatotic liver disease (MASLD) is linked to increased cardiovascular (CV) risks, notably congestive heart failure (CHF). We evaluated the influence of MASLD on CHF and mortality among hospitalized cirrhotic patients.

Methods: We analyzed the National Inpatient Sample from 2016 to 2020, identifying adult cirrhosis patients. We focused on CHF and in-hospital mortality, plus hospital stay length, costs, and discharge status. Propensity score matching created balanced cohorts for comparison. Poisson and logistic regression provided adjusted CHF risks and mortality odds ratios (ORs) for MASLD patients.

Results: Before matching, 4.1% of 672,625 cirrhotic patients had MASLD. Post-matching, each group had 23,161 patients. Patients with MASLD showed higher CHF risk (OR 1.14, 95% CI 1.10-1.21, p<0.001) but lower in-hospital mortality (OR 0.57, 95% CI 0.52-0.63, p<0.01) and decreased costs (median $24,447 vs. $28,630, OR 0.86, 95% CI 0.85-0.87, p<0.001).

Conclusion: In this nationwide study of patients with cirrhosis, MASLD was associated with a higher prevalence of CHF and lower in-patient mortality. These findings mirror the "adiposity paradox" phenomenon, where obese/overweight individuals with cardiometabolic dysfunction may experience less severe or beneficial health outcomes than those with a normal weight. Further investigation is warranted to decode the intricate interplay between MASLD, cirrhosis, CHF, and in-hospital mortality and its clinical practice implications.

## Introduction

Metabolic dysfunction-associated steatotic liver disease (MASLD) is currently the most common cause of chronic liver disease and is defined by the accumulation of fat in more than 5% of hepatocytes, without excessive alcohol consumption (20 grams or more per day) or other known causes of liver disease [1]. The prevalence of MASLD worldwide ranges from 15%-30% based on the utilized methods of estimation, and the prevalence continues to be on the rise, unlike the other common causes of liver disease [2]. Metabolic dysfunction-associated steatotic liver disease is a spectrum of disorders, including hepatic steatosis or fatty liver, metabolic dysfunction-associated steatohepatitis (MASH), and cirrhosis [1]. Metabolic dysfunction-associated steatotic liver disease tends to be asymptomatic until it reaches its advanced stages, and the diagnosis is usually established by using liver enzymes and imaging studies, whereas MASH requires a liver biopsy for confirmation. In recent years, it has become evident that MASLD is a multi-system disorder affecting several extra-hepatic systems [3], and there has been growing evidence and interest in its association with the cardiovascular (CV) system.

The increase in prevalence of MASLD aligns with the rise in metabolic syndrome and obesity. Some researchers consider MASLD a hepatic manifestation of metabolic syndrome, as it shares several risk factors with cardiovascular disease (CVD), such as diabetes, hypertension, hyperlipidemia, obesity, and insulin resistance (IR), suggesting a shared pathogenesis [4]. Multiple studies have demonstrated an association between MASLD and CVD. Initially, the focus of the relationship centered on atherosclerosis and coronary artery disease (CAD), but recent research has also linked MASLD to subtle myocardial dysfunction and arrhythmic manifestations. A large meta-analysis found a significant association between MASLD and a higher prevalence and incidence of cardiovascular events, including CAD, hypertension, and atherosclerosis, though not with all-cause or cardiovascular mortality [5]. Despite substantial research, the relationship between CVD and MASLD remains equivocal, and the association between these conditions may increase or diminish after controlling for the risk factors of metabolic syndrome.

Previous studies suggest a complex and intricate relationship between MASLD, CAD, and cardiac dysfunction. Given the comparability of risk factors, it is difficult to ascertain if MASLD is independently associated with CV events, including CAD and heart failure (HF), or if they are coexisting disorders. There are very few studies conducted on inpatients to study the association between MASLD and CVD-related outcomes. In the inpatient setting, HF imposes a significant burden on healthcare resources and prolongs hospital stays [6]. Heart failure is also the single largest contributor to the annual Medicare budget [6, 7]. Among patients hospitalized for HF, MASLD was shown to be associated with higher rehospitalization rates [8, 9]. On those grounds, this study sought to investigate the relationship between MASLD and CVD outcomes, especially HF, by utilizing a large inpatient database. Given the common risk factors that could confound the relationship between MASLD and CV outcomes, this study relied on a propensity-matched sample (PMS) analysis that ensures comparability between the two groups.

## Materials and methods

Study population and data source

Our study population was obtained from the National (Nationwide) Inpatient Sample (NIS) database. It included patients in the United States hospitalized between the periods of 2016 and 2020. The NIS is the US national database for hospitalized patients, obtained from billing data submitted by hospitals in various states across the US. It provides data for patients’s principal discharge diagnoses during each hospitalization, along with associated secondary diagnoses and procedures performed.

Inclusion and exclusion criteria

Our study focused on adult patients aged 18 and older diagnosed with cirrhosis or MASLD. Patients without a diagnosis of MASLD or under the age of 18 were excluded from the analytic sample. Additionally, we excluded patients with missing information such as age, gender, and race or ethnicity. These variables were accounted for while performing our propensity score matching (PSM) analysis.

Study outcomes

The primary outcome of our study was CHF and in-hospital mortality. Secondary outcomes included hospitalization cost, length of stay (LOS), type of admission, discharge disposition, and median household income. Primary and secondary diagnoses were based on the International Classification of Diseases, Tenth Revision (ICD-10) coding system.

Covariates

Variables such as age, gender, race, ethnicity, and significant comorbid conditions were covariates. The Charlson comorbidity score was used to capture these significant comorbid conditions. Data such as day of admission (weekday or weekend), location and teaching status of the hospital, size of the hospital, and household median income were provided. Also provided were the primary payer source and mortality during hospitalization. This study was exempt from a full institutional review board review, given its study design. The data source, the NIS registry, is a publicly available database, and patients’ information is unidentifiable.

Statistical analysis

Participants were categorized into two groups: patients with the diagnosis of MASLD and patients without the diagnosis of MASLD. We compared and described their clinical and demographic characteristics. Categorical variables were described using counts and proportions. Pearson’s chi-square test was used to test their difference. The Mann-Whitney U test was used to test the differences between the continuous variables, which were presented as a median (interquartile range (IQR)). Adjusted rate and odds ratio (OR) were derived in patients with MASLD compared to those without MASLD using Poisson and logistic regressions.

We performed PSM analyses between patients with MASLD and patients without MASLD, as shown in Table 1. We also performed a 1:1 fixed ratio nearest neighbor matching between patients with MASLD and those without MASLD. We used a 1:1 ratio to minimize bias without sacrificing too much power.

**Table 1:** Characteristics of hospitalized patients with/without MASLD before and after propensity score matching* MASLD: metabolic dysfunction-associated steatotic liver disease; IQR: interquartile range *Weighted counts using Nationwide Inpatient Sample complex survey weights; numbers may not sum to group totals or percentages may not add to 100 owing to the need for rounding. Numbers are rounded to the nearest integral number, and percentages are based on rounded numbers.

Variable	MASLD (N=27,371; 4.1%) n (%)	No MASLD (N=645,254; 95.9%) n (%)	p-value	MASLD (N=23,161; 50.0%) n (%)	No MASLD (N=23,161; 50.0%) n (%)	p-value
Patient characteristics						
Age, median (IQR), y	60 (17)	54 (14)	<0.001	69 (18)	69 (17)	0.99
Gender						
Male	18,393 (67.2)	269,716 (41.8)	<0.001	9,774 (42.2)	9,728 (42.0)	0.67
Female	8,978 (32.8)	375,538 (58.2)		13,387 (57.8)	13,433 (58.0)	
Race/ethnicity						
White	20,802 (76.0)	410,382 (63.6)	<0.001	17,602 (76.0)	17,741 (76.6)	0.45
Black	1,451 (5.3)	79,366 (12.3)		1,227 (5.3)	1,227 (5.3)	
Hispanic	3,750 (13.7)	113,565 (17.6)		3,173 (13.7)	3,080 (13.3)	
Other	1,368 (5.0)	41,941 (6.5)		1,158 (5.0)	1,112 (4.8)	
Charlson Comorbidity Index score						
0	1,697 (6.2)	8,388 (1.3)	<0.001	1,413 (6.1)	1,390 (6.0)	0.55
1-2	2,901 (10.6)	124,534 (19.3)		2,455 (10.6)	2,432 (10.5)	
≥3	22,773 (83.2)	512,332 (79.4)		19,293 (83.3)	19,339 (83.5)	
Comorbidities						
Diabetes	11,797 (43.1)	132,277 (20.5)	<0.001	10,029 (43.3)	9,959 (43.0)	0.4
Hypertension	9,388 (34.3)	173,573 (26.9)	<0.001	8,014 (34.6)	8,083 (34.9)	0.49
Hyperlipidemia	4,297 (15.7)	32,263 (5.0)	<0.001	3,683 (15.9)	3,544 (15.3)	0.1
Type of admission						
Elective admission	3,750 (13.7)	63,880 (9.9)	<0.001	2,270 (9.8)	2,247 (9.7)	0.86
Weekend admission	5,693 (20.8)	143,892 (22.3)	<0.001	4,887 (21.1)	4,864 (21.0)	0.9
Primary payer source						
Private insurance	12,946 (47.3)	225,194 (34.9)	<0.001	10,932 (47.2)	11,117 (48.0)	0.77
Medicaid	3,312 (12.1)	165,830 (25.7)		2,872 (12.4)	2,895 (12.5)	
Medicare	8,677 (31.7)	150,989 (23.4)		7,249 (31.3)	7,296 (31.5)	
Other payment source	1,423 (5.2)	62,590 (9.7)		1,250 (5.4)	1,297 (5.6)	
Self-pay	137 (0.5)	7,743 (1.2)		139 (0.6)	116 (0.5)	
No charge	848 (3.1)	33,553 (5.2)		718 (3.1)	556 (2.4)	
Median household income, $						
<38,999	7,445 (27.2)	216,805 (33.6)	<0.001	6,207 (26.8)	6,091 (26.3)	0.81
39,000-47,999	7,801 (28.5)	167,121 (25.9)		6,277 (27.1)	6,253 (27.0)	
48,000-62,999	6,514 (23.8)	145,182 (22.5)		5,559 (24.0)	5,512 (23.8)	
>63,000	5,611 (20.5)	116,791 (18.1)		5,142 (22.2)	5,304 (22.9)	
Location/teaching status						
Rural	2,983 (10.9)	56,782 (8.8)	<0.001	2,339 (10.1)	2,385 (10.3)	0.98
Urban non-teaching	10,456 (38.2)	258,102 (40.0)		9,102 (39.3)	9,056 (39.1)	
Urban teaching	13,932 (50.9)	330,370 (51.2)		11,719 (50.6)	11,719 (50.6)	
Hospital size						
Small	3,066 (11.2)	70,333 (10.9)	<0.001	2,617 (11.3)	2,571 (11.1)	0.99
Medium	6,131 (22.4)	156,797 (24.3)		5,304 (22.9)	5,327 (23.0)	
Large	18,174 (66.4)	418,125 (64.8)		15,240 (65.8)	15,263 (65.9)	

## Results

Our study included 672,625 hospitalized patients diagnosed with liver cirrhosis upon discharge, of whom 27,371 (4.1%) were diagnosed with MASLD. Table 1 presents the characteristics of hospitalized patients with and without MASLD. Prior to PSM, patients with a diagnosis of MASLD were older (median age 66 ± 17 vs. 54 ± 14 years; p <0.001) and more likely to be male (67.2% vs. 32.8%; p <0.001). They also had higher rates of significant comorbidities (6.2% vs. 1.3%; p <0.001) and exhibited notable racial differences (p <0.001).

The baseline characteristics of the PMS are shown in Table 1**.** The matched patient cohort included 23,161 hospitalized patients diagnosed with MASLD and 23,161 without MASLD. The matched sample was analyzed for age, gender, race, Charlson Comorbidity Index, comorbid conditions, and hospitalization data.

**Figure 1:**
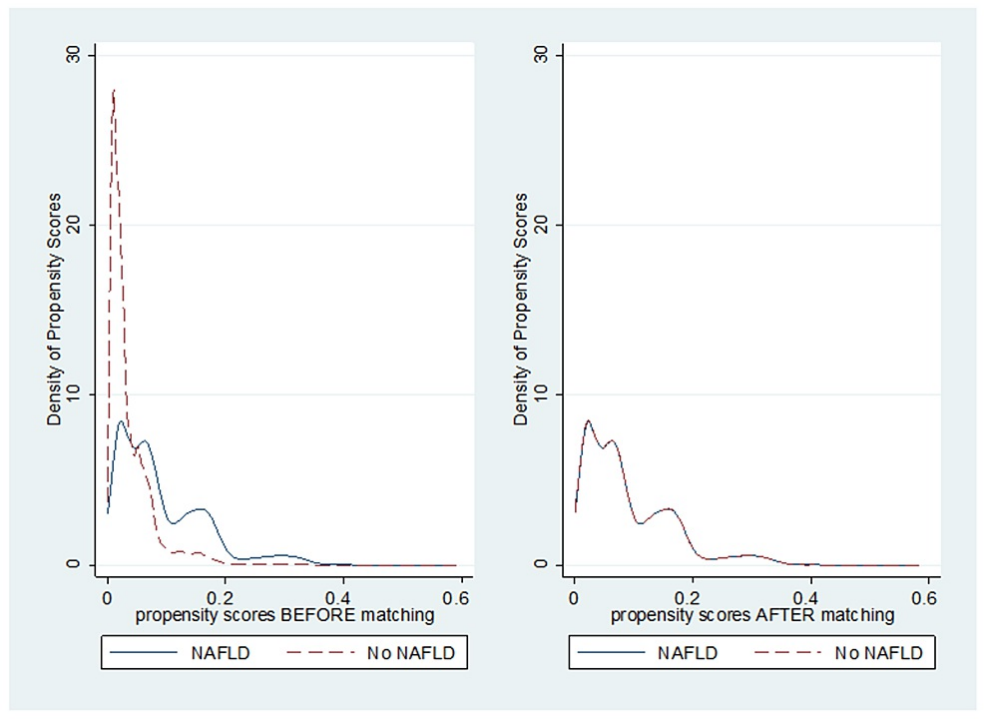
Graph of density propensity score before and after 1:1 fixed ratio nearest neighbor matching NAFLD: non-alcoholic fatty liver disease

**Figure 2:**
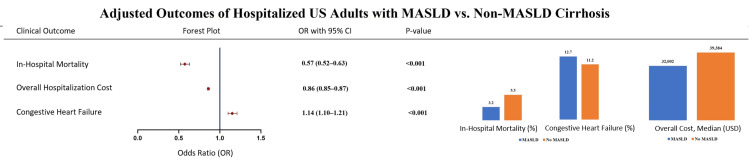
Comparative outcomes of hospitalized U.S. adults with MASLD versus non-MASLD cirrhosis, showing in-hospital mortality, hospitalization costs, and congestive heart failure risks MASLD: metabolic dysfunction-associated steatotic liver disease

Our analysis showed that patients in the MASLD group had a significantly higher risk of congestive heart failure (OR 1.14, 95% confidence interval (CI) 1.10-1.21, p <0.001). However, they paradoxically experienced a lower risk of in-hospital mortality (OR 0.57, 95% CI 0.52-0.63, p <0.001) and overall reduced hospitalization costs (median $24,447 vs. $28,630; OR 0.86, 95% CI 0.85-0.87, p <0.001) compared to those without MASLD. These findings are presented in Table 2 and Figure 1. Finally, Figure 2 represents a central illustration that summarizes the key findings presented in the results section.

**Table 2:** Propensity score–matched outcomes between patients with/without MASLD MASLD: metabolic dysfunction-associated steatotic liver disease; OR: odds ratio, using conditional logistic regression; CI: confidence interval; LOS: length of stay; **incident risk ratios, using Poisson regression

Outcome	MASLD (N=23,161; 50.0%)	No MASLD (N=23,161; 50.0%)	OR (95% CI)	p-value
Congestive heart failure, no. (%)	2,940 (12.7)	2,606 (11.2)	1.14 (1.10-1.21)	<0.001
LOS, median (IQR), d	4 (5)	4 (5)	#	0.99
Overall cost, median (IQR), USD, $	24,447 (32,002)	28,630 (39,384)	0.86 (0.85-0.87)^**^	<0.001
In-hospital mortality, No. (%)	740 (3.2)	1,268 (5.5)	0.57 (0.52-0.63)	<0.001
Discharge disposition				
Routine discharge or home self-care, no. (%)	15,151 (65.4)	13,584 (58.6)	1.33 (1.28-1.38)	<0.001
Short-term hospital, no. (%)	874 (3.8)	791 (3.4)	1.10 (1.01-1.22)	0.04
Skilled nursing facility, no. (%)	2,959 (12.8)	4,038 (17.4)	0.69 (0.65-0.73)	<0.001
Home healthcare, no. (%)	3,200 (13.8)	2,991 (12.9)	1.10 (1.02-1.14)	0.01

## Discussion

The aim of this study was to examine the association between MASLD and CV outcomes in hospitalized patients, specifically the risk of CHF, as well as in-hospital mortality and hospitalization costs. Using the United States NIS data, patients diagnosed with MASLD were significantly associated with a higher risk of CHF. However, these patients interestingly had a lower risk of in-hospital mortality with an OR of 0.57 and a reduced cost of hospitalization when compared to patients without MASLD after PSM. The novelty of our study lies in its large-scale analysis and its contribution to the limited literature on this topic by confirming the elevated risk of CHF in MASLD patients while revealing unexpected outcomes such as reduced in-hospital mortality and hospitalization costs.

Metabolic dysfunction-associated steatotic liver disease and CVD have several molecular mechanisms in common, which may be helpful in explaining the relationship between the two. Oxidative stress is one common underlying factor in the pathogenesis of both MASLD and CVD. Fatty liver may contribute to CVD by increasing the expression and release of pro-inflammatory and procoagulant cytokines [10]. Endothelial dysfunction occurs very early in the process of atherosclerosis. Studies have shown a direct association between MASLD and endothelium-dependent flow-mediated dilation, which is worse in MASH compared to simple hepatic steatosis [11]. It is important to consider that the overall sicker profile of the non-MASLD cirrhosis group could also explain some of the elevated cardiovascular risks associated with MASLD, possibly through mechanisms not directly related to the liver pathology itself. This raises the question of a dose-response relationship between the severity of MASLD and CVD risk. Coronary angiogram-based studies have also shown the association between MASLD and CAD, independent of their common risk factors [12,13]. Coronary arterial calcification demonstrated by computed tomography (CT) has been shown to be associated with MASLD [14], with one study proving a relationship with vulnerable plaques rather than coronary stenosis [15]. Carotid artery intimal media thickness (IMT) is a marker of subclinical atherosclerosis, independent of the usual CAD risk factors. The association between fatty liver and IMT has been proven in prior studies [16,17]. Higher values of fatty liver index (FLI) have been shown to be associated with increased IMT and CAD [18]. The histopathological severity of MASLD has been shown to be significantly associated with carotid atherosclerosis [19]. Another study showed that the association was stronger for MASLD with carotid atherosclerotic plaques than only IMT [20]. Insulin resistance is central to the etiopathogenesis of MASLD, atherosclerosis, and metabolic syndrome. Hepatic fat itself, rather than visceral adipose tissue, has been shown to be an independent predictor of IR [21], not only in the liver but also in other important organs like skeletal muscle and adipose tissue. Hence, MASLD may be a powerful stimulus for worsening IR, leading to an overproduction of fatty acids that contribute to atherosclerosis.

While most of the studies associating MASLD and CV risk are based on the theories of atherosclerosis and CAD, there has been a lot of recent focus on the effect of a fatty liver on cardiac function and remodeling of the myocardium. A study was conducted in patients with diabetes and MASLD, which revealed these patients had early features of left ventricular (LV) diastolic dysfunction, though the LV morphology and ejection fraction were unaffected [22]. Another study also showed that MASLD was associated with impairment of LV function and several markers of diastolic dysfunction that persisted after adjusting for BMI [23]. They also showed increased LV mass, end-diastolic LV volume, and left atrial (LA) volume index, which are all markers of the severity of LV diastolic dysfunction and point towards subclinical LV remodeling in these patients. The association persisted, albeit with some attenuation when adjusted for markers of obesity and other risk factors for HF. Later studies also confirmed the above findings that MASLD could be an early marker of LV remodeling and diminishing cardiac function [24,25].

The association between MASLD and CVD has been shown in prior clinical studies [19, 26]. A prospective cohort study in patients undergoing coronary angiograms identified a significant association between fatty liver demonstrated on ultrasound and CAD; however, mortality was unaffected [27]. Another study found a significant relationship between fatty liver and future CVD risk when adjusted for other covariates but not for insulin sensitivity [28]. Friedrich-Rust et al. performed a study on the relationship between the severity of CAD as determined by the coronary angiogram and the severity of MASLD assessed by transient elastography and controlled attenuation parameters. They found that patients with clinically relevant CAD were more likely to have MASLD, but there was no association with advanced fibrosis [29]. Mellinger et al. studied the relationship between fatty liver and CVD outcomes using follow-up data from the Framingham Heart Study. They found that hepatic steatosis was associated significantly with subclinical atherosclerosis, including coronary calcifications, in a multivariate-adjusted model but not with clinical CAD [30]. Two previous studies based on the National Health and Nutrition Examination Survey (NHANES) data compared the prevalence of CVD and mortality related to CVD in patients with and without MASLD after adjusting for the risk factors. They showed that MASLD patients are more likely to have CVD; however, there was no difference in CV mortality [31,32].

There is very limited literature on MASLD and the risk of HF, especially in the inpatient population. Valbusa et al. studied a cohort of hospital readmissions for acute HF, its relationship with MASLD, and the severity of liver disease. They showed that MASLD was associated with an increased risk of readmission for all causes and HF, and these were higher for patients with advanced fibrosis [9]. Another recent study looked at the severity of fibrosis based on the MASLD fibrosis score (MFS) and adverse CV outcomes in patients with chronic HF. They found that with the increase in MFS, there were more adverse CV outcomes and patients with higher MFS were associated with poor New York Heart Association (NYHA) functional class and worse clinical outcomes [33]. There are various plausible mechanisms linking the fatty liver and HF. Firstly, this could be due to the effect on coronary arteries, leading to the worsening of CAD. Subtle effects on myocardial remodeling and function, as demonstrated in some prior studies, could play a significant role. In addition, MASLD and its severity have been shown to be associated with an increased risk of atrial fibrillation and conduction defects, which could also result in cardiac decompensation [34-37].

As shown, the available literature on the relationship between MASLD and CV outcomes has yielded heterogeneous results with very minimal data on hospitalized patients. In studying the association between MASLD and CV events, including HF, multiple shared risk factors can result in confounding results. Hence, in our study, we performed a PMS analysis of the two comparison groups, which took care of the underlying differences between them. The odds of HF in hospitalized patients with MASLD are 1.14 times higher than those without. Considering the increasing prevalence of MASLD and the burden of HF on our medical resources, this is an area that needs to be further investigated in prospective studies.

Strengths and limitations

Our study had inherent limitations due to its observational and retrospective design. For instance, the database did not allow us to assess long-term and post-discharge outcomes, limiting our ability to fully understand the impact of MASLD on cardiovascular outcomes over time. Additionally, potential errors in ICD-10 coding could have introduced bias or inaccuracies in the data, which we could not account for. Given that the data were sourced from multiple centers, variations in the criteria for diagnosing MASLD may have occurred, and severity data on MASLD and fibrosis scores were not available for all patients. While the adiposity paradox might explain some of the unexpected findings in our study, such as the lower in-hospital mortality and reduced hospitalization costs in MASLD patients, it is important to consider that other underlying physiological or clinical factors specific to the etiology of cirrhosis in different patient groups could also significantly influence these outcomes. Factors such as potential differences in the management or monitoring of MASLD patients during hospitalization, variations in the severity of liver disease among MASLD patients, and differences in the etiology of cirrhosis may have influenced our findings. Additionally, the limitations of our study, including reliance on ICD-10 coding and the lack of comprehensive data on MASLD severity and fibrosis scores, should be taken into account when interpreting our results.

Despite these limitations, our study possesses several notable strengths. The large sample size enhances the generalizability of our findings to the entire United States population. Additionally, the use of a propensity score-matched cohort helps minimize confounding variables, providing a more robust and accurate assessment of the association between MASLD and cardiovascular outcomes. This approach strengthens the validity of our results and the conclusions drawn from the data. Nonetheless, future prospective studies are needed to address these limitations and further explore the relationship between MASLD and cardiovascular events.

Future research

Given the complexity and multifactorial nature of the relationship between MASLD and cardiovascular outcomes, future research should focus on prospective, multicenter studies that can provide a more comprehensive understanding of the long-term effects of MASLD on heart health. These studies should aim to standardize the diagnostic criteria for MASLD and include detailed assessments of fibrosis severity and liver function to better delineate the progression and impact of the disease. In addition, advanced imaging techniques such as cardiac magnetic resonance imaging and echocardiography could offer deeper insights into the cardiac remodeling and dysfunction associated with MASLD. Investigations into the molecular pathways linking MASLD and CVDs, including inflammation, oxidative stress, and IR, would also be valuable. Ultimately, well-designed interventional trials may identify effective strategies for managing MASLD and mitigating its cardiovascular complications, thereby improving patient outcomes and quality of life.

## Conclusions

Our study has a myriad of therapeutic implications. It reinforces the existing knowledge that MASLD is a multi-system disease that merits a more elaborate and multidisciplinary assessment of the patient. In hospitalized patients who have a diagnosis of MASLD, there should be a detailed screening of the CV status and cautious monitoring of the fluid status. These measures can potentially reduce the risk of HF or other adverse CV outcomes. It might also be reasonable to proactively screen patients with MASLD for CVD and CV risk factors. This can result in the timely implementation of lifestyle modifications or the introduction of medications to control these factors even before they are clinically apparent. Prudent surveillance, risk factor stratification, and early intervention in these patients can reduce the risks implicated in developing HF whether or not patients with CVD and/or HF need to be screened for MASLD. The most commonly utilized imaging modality to corroborate the diagnosis of MASLD is ultrasonography, which has a high specificity but lower sensitivity, especially when the hepatic fat content is low. Magnetic resonance spectroscopy is considered the gold standard imaging technique for quantification of liver fat content; however, it is expensive and not easily assessable. Pending the wide availability of Magnetic resonance spectroscopy, history, liver functions, and ultrasound imaging can be used to diagnose MASLD. 
